# Agreement and consistency in the triaging of musculoskeletal primary care referrals by vetting clinicians using a knowledge-based triage tool

**DOI:** 10.1017/S1463423623000361

**Published:** 2023-10-26

**Authors:** F. M. Shorthouse, N. Griffin, C. McNicholas, N. Spahr, G. Jones

**Affiliations:** 1 Musculoskeletal Physiotherapy Service, Guys and St Thomas’ NHS Foundation Trust, Westminster Bridge Road, SE1 7EH, UK; 2 Physiotherapy Service, Guys and St Thomas’ NHS Foundation Trust, Westminster Bridge Road, SE1 7EH, UK

**Keywords:** triage, musculoskeletal, primary care

## Abstract

**Background:**

Primary care referrals received by secondary care services are vetted or triaged to pathways best suited for patients’ needs. If knowledge-based triaging is used by vetting clinicians, accuracy is required to avoid incorrect decisions being made. With limited evidence to support best practice, we aimed to evaluate consistency across vetting clinicians’ decisions and their agreement with a criterion decision.

**Methods:**

Twenty-nine trained vetting clinicians (18 female) representative of pay grades independently triaged five musculoskeletal physiotherapy referral cases into one of 10 decisions using an internally developed triage tool. Agreement across clinicians’ decisions between and within cases was assessed using Fleiss’s kappa overall and within pay grade. Proportions of triage decisions consistent with criterion decisions were assessed using Cochran’s *Q* test.

**Results:**

Clinician agreement was fair for all cases (*κ* = 0.385) irrespective of pay grade but varied within clinical cases (*κ* = −0.014–0.786). Proportions of correct triage decisions were significantly different across cases [*Q*(4) = 33.80, *P* < 0.001] ranging from 17% to 83%.

**Conclusions:**

Agreement and consistency in decisions were variable using the tool. Ensuring referrer information is accurate is vital, as is developing, automating and auditing standards for certain referrals with clear pathways. But we argue that variable vetting outcomes might represent healthy pathway abundance and should not simply be automated in response to perceived inefficiencies.

## Background

Patients present to a general practitioner (GP) with a wide range of complaints. If secondary or tertiary care is required from a healthcare service, then a referral is made with the relevant information to prioritise the patients’ needs (Department of Health (DH), 2015). It is estimated that 30% of GP consultations are for musculoskeletal (MSK)-related conditions (Department of Health (DH), 2006) which are often referred to physiotherapy in secondary care. Primary care referrals to hospitals in the UK are typically administered by GPs using the e-Referral service (e-Rs, NHS Digital & BJSS plc., Leeds, UK (BJSS plc, 2021)) which recently replaced the Choose and Book system, first introduced in 2004. Irrespective of referral service systems a GP might use, it is vital the referral is triaged to ensure the appropriate service pathway is selected. This is only achieved if the receiving service can be assured that its referral vetting, or triaging processes, lead to optimal clinical decision making.

Clinical decision tools are designed to augment clinical decision-making processes. The typology spectrum of clinical decision tools ranges from paper-based reminder cue sheets to electronic diagnostic/prognostic probabilistic modelling (Liu *et al.*, 2006) whose development is likely to continue rapidly (Middleton *et al.*, 2016). Irrespective of typology, tools are often classified as either knowledge based (where firstly IF-THEN logic rules are specified and then the system retrieves knowledge to evaluate the rule) or non-knowledge based (where decisions based on data sources are induced by artificial intelligence, machine learning or statistical pattern recognition) (Sutton *et al.*, 2020). While developments are aimed to better augment shared clinical decision making between medical/paramedical professionals and patients during consultations, clinical decision tools have also been utilised upstream to better augment the triaging of referrals to services by vetting clinicians. Triage tools are designed to ensure that referrals include all relevant information (and advise re-referral or rejection if not), and that the patients are triaged to a specialist service with the prerequisite knowledge and skills to assess and manage their needs (Cummins *et al.*, 2015, Scottish Government, 2018, Deloitte LLP, 2019).

### Local context

In our large, inner-city hospital MSK adult physiotherapy service, we have adopted a referral triaging system that incorporates a knowledge-based clinical decision support tool (Sutton *et al.*, 2020). All MSK physiotherapists are expected to triage referrals after attendance at a standardised training session to ensure observed competence and then undergo a familiarisation period where triaging is undertaken jointly with a more knowledgeable other (Vygotsky, 1978) to address confidence (Gottlieb *et al.*, 2022). When triaging, a trained clinician is first triggered by the triage tool text (see supplemental material/Appendix 1) to assign the case to a major joint or body segment category typically treated in secondary MSK practice (spine, hip, knee, shoulder/elbow, wrist/hand, foot/ankle). Then, the tool specifies IF-THEN logic rules for the vetting clinician who is then triggered to scrutinise data from the referral in order to evaluate the rules. This in turn produces an action (e.g. reject, or refer to service external to physiotherapy) or an output (triage the case into one of the eight adult MSK physiotherapy service pathways available).

The tool also includes cross-category text-box alerts which act as reminders to clinicians to include important contextual factors in their decision making. These include referrals for a paediatric rather than an adult case, evidence (or suspicion) of serious pathology, acute fracture (National Institute for Clinical and Health Excellence (NICE), 2022), or a systemic inflammatory condition, or the referral is incomplete or a duplicate. Clinicians are reminded to consider actions: either return the referral (reject it), discuss with the referrer or hand the referral off to other agreed secondary care pathways. There is another text-box embedded in the tool that informs vetting clinicians that advice from experienced expert colleagues should be sought in the case of suspicious or clinically ambiguous referrals. Lastly, the tool includes IF-THEN-OTHERWISE rules triggering clinicians to determine whether the referral is urgent or otherwise routine and IF-THEN rules to determine whether patients should be offered on-line or face-to-face appointments first.

The rules specified in our tool, referred to locally as a vetting grid, were internally designed after engagement with our medical and surgical partners (e.g. rheumatology and orthopaedics) and other therapy services. The grid was first developed in 2015 and last modified in 2021. The vetting grid has iteratively evolved to reflect new evidence-based, practice-based or patient-directed guidance from local service development projects (Osheroff *et al.*, 2012). It does however remain a paper-based guide (albeit digitised to be viewed within existing electronic note applications). As such, it might not be sophisticated enough to ensure the cognitive support the tool provides (Middleton *et al.*, 2016) is comprehensive as would be the case if, for example, an automated knowledge tree approach was adopted. While knowledge trees are by no means new (Shiffman and Greenes, 1994), and are still being developed in, for example, breast cancer clinical decision making (Hendriks *et al.*, 2019), it is possible that local MSK triaging decisions may be more efficient if introduced.

The majority of MSK referrals are received from GP practices registered in two local metropolitan boroughs with a combined resident population of 643 200 (2017 estimate (Greater London Authority (GLA), 2021)), as well as referrals from other secondary care and tertiary services. In the calendar year 2020–2021, our MSK department received a mean (±SD) of 2,125 (±425) GP referrals per month. This equates to an average rate of 51 referrals every day, making it one of the busiest outpatient services in the local NHS Trust.

We have designed our cognitive aide vetting grid as a means of efficiently and competently dealing with a large volume of referrals. As yet, there is relatively little published data on the evaluation of MSK referral triaging processes in secondary care. One previous study in an ENT outpatient service observed good referral vetting consistency between otolaryngology consultants and nursing staff (Hathorn *et al.*, 2009). However, the scale of referral rates and vetting options was relatively modest in this study compared with our MSK service, less than 20 referrals per month and only three vetting options (urgent, soon or routine).

We therefore wished to find out whether our referral triaging tool was precise (do all clinicians vet to the same service?) and accurate (are referrals triaged to appropriate services?). We wished to determine how well physiotherapy clinicians agreed on their triaging decisions using the vetting grid, whether the type of referral or clinical experience influenced the agreement and whether triaging decisions made on typical patient referral types were consistent with criterion decisions. We envision that these assessments will support the development of the triaging tool, inform clinician training and be a basis with which to evaluate clinical outcomes of the clinical pathways’ patients are vetted into.

## Methods

**Figure 1.**
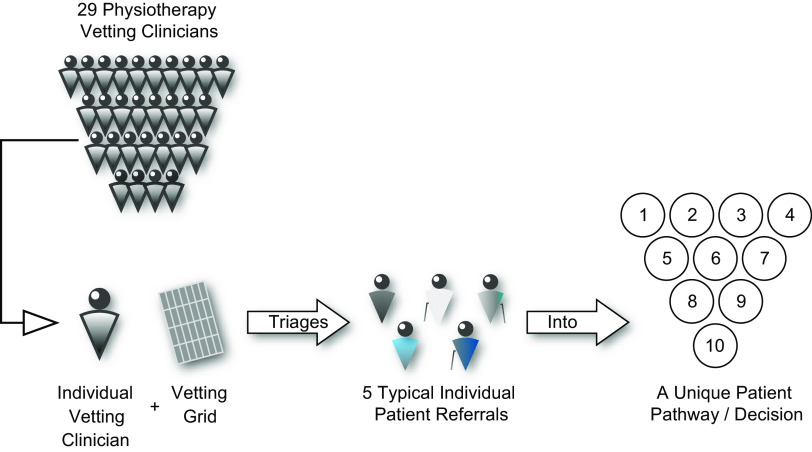
Participant triaging schematic for vetting physiotherapy clinicians (*n* = 29) each independently triaged 5 predetermined referrals; presented in a predetermined standardised order into one of 10 predetermined decisions pathways using an established vetting grid tool (see Appendix 1).

This was a rater comparison study ethically approved by the local NHS Foundation Trust directorate governance committee (ref: 54 321) which provides oversight in compliance with the Helsinki Declaration (General Assembly of the World Medical Association, 2014). All eligible qualified MSK physiotherapists (*n* = 50; 26 Band 6, 10 Band 7, 14 Band 8 (Agenda for Change (AFC) pay band where numbers increase with seniority (NHS Employers, 2010)) familiar with and trained in the use of our vetting grid tool were invited to participate via an email invitation (between April and June 2021) and 29 accepted. Participants were provided with a private clinic room (to prevent conferring) to independently triage 5 anonymised MSK patient case referrals each into one of 10 predetermined action or output triage decisions (Fig. 1) using the existing vetting grid used in clinical practice (supplemental material/Appendix 1) presented on a laptop using proprietary software (SurveyMonkey, Momentive Inc., San Mateo, CA.).

The five patient cases represented a consecutive sample of every 5th referral received on a day in June 2021 and was undertaken by a blinded non-clinical administrative staff member. The cases were then randomly sequenced *a priori* by one of the authors (FS) and each vetting clinician triaged then in the same order. The patient cases reflected typical referrals to the department (older-adult non-specific knee pain (knee), adult non-specific neck pain (neck), adult hand trauma (hand), non-specific hip pain (hip), paediatric limb pain/dysfunction (paed)). Triage decisions were constrained to 10 possible outcomes used clinically within the MSK service: 2 actions (reject or onward referral) and 8 outputs representing MSK clinical pathways (see Table 1).

**Table 1.** Summary of individual Fleiss’s kappas to assess triage decisions for each decision pathway

Triage decision		κ	(95%CI)	*P*
1	Back Clinic	0.022	(0.021 to 0.024)	0.317
2	F2F MCATS Clinic	0.339	(0.337 to 0.340)	<0.001
3	F2F Physiotherapy Clinic	0.042	(0.040 to 0.043)	0.061
4	Hand Clinic	0.786	(0.785 to 0.787)	<0.001
5	Integrated Clinic	0.040	(0.039 to 0.041)	0.072
6	Remote MCATS Clinic	−0.014	(−0.015 to −0.013)	0.529
7	Remote Physiotherapy Clinic	0.086	(0.085 to 0.088)	<0.001
8	Onward Referral	0.613	(0.612 to 0.615)	<0.001
9	Reject Referral	0.474	(0.473 to 0.476)	<0.001
10	PFJ Clinic	–	–	–

The kappa statistic and its 95%CI are how along with the probability the kappa differs from zero.

Triage decision included eight physiotherapy clinics some of which are offered face-to-face (F2F) or remotely (on-line) (Back Clinic [a Band 8 led clinic any patient with back pain]; MCATS clinic [Assessment clinic with Band 8]; General Clinic [one to one Physiotherapy assessment]; Hand Clinic [specialist hand therapist clinic]; Integrated Clinic [Band 8 led clinic for any patient with a complex peripheral joint complaint]; PFJ Clinic [Band 8 led clinic with patients with suspected PFJ pain]) and decisions to either reject (return to GP) or refer-on F2F = face-to-face; MCATS = Musculoskeletal Clinical Assessment and Treatment Service; PFJ = Patellofemoral Joint clinic.

The agreement across all vetting clinicians’ decisions from 10 possible outcomes across 5 MSK patient referrals was assessed using Fleiss’s kappa with individual kappas used to assess agreement for each vetting decision pathway. Fleiss’s kappas were also calculated to assess the triage decision agreement across each clinician at the same pay banding. Interpretations of the strength of agreement were based on published guidelines (Landis and Koch, 1977, Altman, 1999).

Triage decisions were compared with a criterion for each referral established by an MSK clinician blinded to the study (18 years experienced, AFC band 8a). The percentage of correct triage decisions by the vetting clinicians (i.e. consistent with the criterion decision) within each of the five patient referral types was assessed using a Cochran’s *Q* test (Cochran, 1950). Sample size was adequate to use the χ^2^-distribution approximation (Tate and Brown, 1970). Pairwise comparisons were undertaken using Dunn’s (Dunn, 1964) procedure (Bonferroni correction for multiple comparisons) with adjusted *P* values presented if there was a significant difference. For all statistical tests (SPSS v26, IBM Corp, Armonk, NY, USA), statistical significance was assumed if *P* ≤ 0.05.

## Results

Twenty-nine physiotherapists (18 (62%) female, 11 (38%) male) agreed to participate. Their professional experience (mean (±SD) years) per pay banding (AFC Band 6-8a) was 2.8(±0.7) years at Band 6 (*n* = 12), 4.1(±2.7) years at Band 7 (*n* = 6) and 11.4 (±4.5) years at Band 8a (*n* = 11). This was reflective of the department, which at the time had 26 band 6, 10 band 7 and 14 band 8 staff.

There was fair agreement across all clinicians in their triage decisions [κ (95%CI) = 0.385 (0.384 to 0.386), *P* < 0.001] irrespective of the criterion decision. There was no evidence that this was influenced by pay grade: [Band 6, κ(95%CI) = 0.348 (0.347 to 0.350), *P* < 0.001], [Band 7, κ(95%CI) = 0.394 (0.391 to 0.397), *P* < 0.001], [Band 8a, κ(95%CI) = 0.387 (0.386 to 0.389), *P* < 0.001].

Individual kappas revealed there was good agreement across all clinicians triaging patients into the hand clinic (*κ* = 0.786) or for onward referral (*κ* = 0.613) with the remainder being either fair or poor agreement (Table 1).

The percentage of triage decisions consistent with the criterion decision was statistically different across the five patient referral types [*Q* (4) = 33.80, *P* < 0.001]. Compared to the low proportion of correct decisions with the knee referral type (17%), there was a statistically higher proportion for the neck (29%, *P* < 0.001), hand (83%, *P* < 0.001), hip (79%, *P* < 0.001) and paed (59%, *P* = 0.015) referral types. There were no statistically significant differences in proportions in any other pairwise comparisons (Table 2).

**Table 2.**
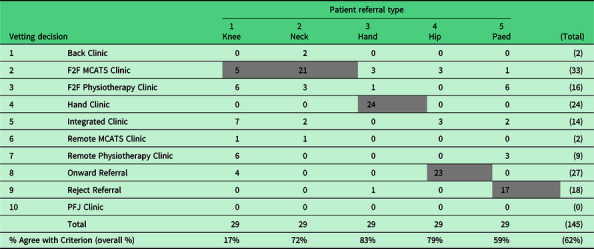
Summary of all vetting clinicians’ triage decisions per patient referral type

Numbers represent how many of the 29 physiotherapists selected each triage decision per referral type. Shaded cells represent the criterion triage decision for each referral type.

Triage decision included 8 physiotherapy clinics some of which are offered face-to-face (F2F) or remotely (on-line) (Back Clinic [a Band 8 led clinic for any patient with back pain]; MCATS clinic [Assessment clinic with Band 8]; General Clinic [one to one Physiotherapy assessment]; Hand Clinic [specialist hand therapist clinic]; Integrated Clinic [Band 8 led clinic for any patient with a complex peripheral joint complaint]; PFJ Clinic [Band 8 led clinic with patients with suspected PFJ pain]) and decisions to either reject (return to GP) or refer-on F2F = face-to-face; MCATS = Musculoskeletal Clinical Assessment and Treatment Service; PFJ = Patellofemoral Joint clinic.

## Discussion

The purpose of this study was to determine how well musculoskeletal physiotherapy clinicians agree on their triaging decisions using our locally developed cognitive aide vetting grid. In addition, we wished to discover whether the type of referral or clinical experience influences the agreement and determine whether triaging decisions made on typical patient referral types agree with criterion decisions. We wished to gather this information to further develop the triaging tool itself and also inform training for vetting clinicians and will by virtue of assuring of the vetting processes be a basis with which to evaluate the clinical outcomes of the clinical pathways patients are vetted into.

### Main findings

Twenty-nine trained physiotherapists were instructed to independently triage five typical MSK patient case referrals using an established knowledge-based vetting grid tool used as a cognitive aide into one of 10 possible outcomes. We examined how much their decisions agreed overall and by pay grade, and how consistent their decisions were with respect to a criterion decision. Overall agreement was merely fair which reflects the variability of triage decisions. However, our observations across 29 physiotherapists representing the spectrum of pay grades supported the view that agreement was not influenced by pay banding. We believe that this reflects the competency of our training methods in teaching staff across all levels in how to use the grid. Agreement was best if referral vetting decision outcomes included a sub-speciality (hands) or were actions (onward or reject referral) suggesting that information received in these cases conferred more precise interpretation using the vetting grid tool.

These triage decisions are therefore either uncontentious enough to be adequately triaged using the vetting grid or could be candidates for automatic automation if GP referrals are expected to consist of standardised, unambiguous data. There is support to use more digital decision systems in the NHS which would bypass human error and is in keeping with both an emphasis on primary care referrers maximising meaningful data in e-Rs referrals to physiotherapy (Gandhi *et al.*, 2000) and with NHS England’s improved productivity vision (NHS England, 2015).

Triaging was significantly worse (17%) when vetting an older-adult knee pain referral appropriate for F2F MCATS (face to face Musculoskeletal Clinical Assessment and Treatment Service). Decisions for this patient were highly variable. There are several intersecting factors which might explain this observation. Firstly, a reluctance to vet to MCATTS by more junior staff might exist because MCATTS is staffed by senior colleagues with implicit power relationships (Latash, 2012). However, the neck patient was appropriately triaged to F2F MCATTS significantly more consistently (72%) with the criterion decision than the older adult with knee pain. This suggests that power perceptions were probably not factors featuring in the triage decision for the older patient we observed. Secondly, this case was triaged, legitimately, to a number of various pathway options available (face-to-face physiotherapy, integrated clinic, remote MCATTS, remote physiotherapy or onward referral) because vetting clinicians perceived the patient’s needs could be met by more than one definitive pathway based on the information provided in the referral and the IF-THEN rules inherent in the vetting grid. To limit cross-contamination among the sample, a private space was provided for participants to triage the referrals in. But in so doing, we might have unintentionally constrained participants’ ability to seek senior or peer expert consultation if the referral was interpreted as ambiguous or complex. Given that the vetting grid alerts vetting clinicians to seek expert opinion if there is ambiguity, we acknowledge this as a weakness in our design – as was opting to not collect data about whether participants would have sought second opinions. Thirdly, it is possible triage decisions were variable because this case was triaged without due diligence secondary to prejudice, be it unintentional or not. In this case, prejudice towards age might be possible seeing that ageism certainly exists in healthcare professions (Nelson, 2005). But it is impossible to expand further on this sentiment seeing as we neither specified observing prejudice during the triaging process in our aims nor attempted to measure it. A more likely explanation is that the older-person referral was complex and the variation observed in triage decisions was a symptom of that complexity. Clinicians’ opinions about where cases could be referred to for the patient’s best interests were probably variable irrespective of whether any ageism influenced the decision. Older patients with any pathology are often burdened with multimorbidity (such as hypertension, hypercholesterolaemia and diabetes to name a few) which is associated with an increase in clinical and social complexity (Jones *et al.*, 2017). Examining the factors that influence individual decision making when triaging and minimising them is an area that would benefit from further research. Whether this complexity was or was not alluded to in the referral, and how much vetting clinicians synthesised that information in their decision probably modulated the amount of variability in decisions seen. The large variability in this patient case is therefore likely to be at the intersection of the patient type of referral (older-person) and the quality of the information received in the referral (amount of contextual medical and social morbidity data).

Even if referrals for complex cases included impeccable data that were interpreted flawlessly by the receiving service, we must not automatically assume that a reduction of clinical pathways available to treat the patient is deleterious or inefficient. After all, one person’s redundancy is another’s abundancy (Latash, 2012), and if this were accepted, then the vetting grid tool could be amended to include IF-THEN-OR rules. Yet the fact that physiotherapists administer their own referrals is probably inefficient in the eyes of lean-pathway advocates who would instead endorse a third party or information technology solution acting as a referral management service (RMS). RMSs are not a panacea though. Assessment of stakeholders’ perceptions of an RMS was negative in a qualitative study of GPs referring to secondary care consultant clinics via an RMS (Dew and Wilkes, 2019). Perceived negativity included the RMS creating a barrier between GPs and consultants therefore affecting opportune clinician-to-clinician contextual discourse for the benefit of individual patients.

Our data suggest that referrals of the type where vetting variability is low may be excellent candidates for information technology-based automation, offering the least error in predictability for any system and improving efficiency in time for clinicians and the patient. This has been shown to improve communication in transitions between secondary care episodes and discharge to community care for example (Scotten *et al.*, 2015). But it is incorrect at this stage to wholly interpret high variability in our vetting decisions negatively, and reduce all vetting decisions to an automated RMS. Instead, variable pathway options could be considered aspirational as a legacy of abundant and diverse clinical and communication expertise in a service. This is not inconsequential because it engenders timely interaction across sub-specialties in the system, reduces total hand-offs (bureaucratic inter and intra-service conveyance) and at the same time improves the efficiency of hand-offs that are necessary (Benham-Hutchins and Effken, 2010). Instead of a broad-brush RMS solution then, an assessment of what effect variable vetting decisions have on clinical consequences downstream in the system is indicated to determine if abundancy (some variability in vetting) is objectively palatable.

### Limitations

We acknowledge other weaknesses in our methods. Firstly, physiotherapists self-selected whether to participate or not which could have introduced bias. A cluster randomised sample from all competent vetting clinicians accounting for decline to participate rates, and ensuring equal representations among all pay bands, would have been more robust. Secondly, while using criterion decisions based on a blinded member of staff was valid, basing the decision on the consensus of >1 expert would have increased the validity. Thirdly, we could with more resources have included a larger number of cases, but we adopted a pragmatic real-world approach and aimed to limit deviation from essential clinical tasks and maximise participation by adopting five cases in this project.

## Conclusion

Triaging decision making in a large MSK physiotherapy service is a variable dependent on the type of patient referral, the information it confers and the pathway to which the referral is triaged to. Our results indicate we should tailor further work to improve processes by firstly ensuring referrer information is accurate and meaningful, and secondly develop, automate and audit standards for particular types of referrals that have clear pathway routes. Lastly, variability in vetting outcomes might represent a healthy abundance of pathways and skill rather than automatically reflect inherent inefficiency. Therefore, in future work we will also determine whether relative efficiency (hand-offs management), treatment time and clinical outcomes are equivalent or differ between patients vetted to different pathways.

## Supplementary material

For supplementary material accompanying this paper visit http://doi.org/10.1017/S1463423623000361. click here to view supplementary material
