# Supplementary material for: Agreement and consistency in the triaging of musculoskeletal primary care referrals by vetting clinicians using a knowledge-based triage tool
Source: Prim Health Care Res Dev. 2023 Oct 26;24:e63. doi: 10.1017/S1463423623000361 (PMC10790367; doi:10.1017/S1463423623000361)
Supplement: Supplementary file 1 [file S1463423623000361sup001.pdf]

Spine

Physiotherapy

1. MLBP no previous physio OR previous good response
2. Chronic LBP (no previous physio OR previous good response)
3. LBP with radicular leg pain or LxSp radiculopathy < 6/52
4. Mechanical thoracic or cervical pain (no previous physio OR previous good response)
5. Neck pain with radicular arm pain or CxSp radiculopathy <6/52
6. WAD (no neurology)

(Items 1–3: BACK CLINIC if GSTT)

MCATTS:

1. MLBP & failed conservative management
2. Chronic LBP & failed conservative management
3. LBP with radicular leg pain or LxSp radiculopathy > 6/52
4. Lumbar canal stenosis on imaging
5. Mechanical thoracic or cervical pain (failed conservative management
2. Neck pain with radicular arm pain or CxSp radiculopathy >6/52
4. Thoracic or cervical stenosis on imaging
5. WAD with neurology
6. Scoliosis - painful without worsening deformity or in absence of MRI in past 6/12
7. Previous surgery - if outside Orthopaedic criteria

(Items 1–4: BACK CLINIC if GSTT)

Cauda Equina Syndrome

*****Reject and contact GP urgently to advise A & E Referral*****

- Saddle anaesthesia
- Urinary retention
- Faecal Incontinence

Orthopaedics [Neurosurgery at KCH]
*****Requires MRI in past 6/12*****

1. Scoliosis [*<u>ONLY</u> to GSTT <u>NOT</u> KCH]*
   - Adolescent Idiopathic
   - Worsening deformity
2. Previous spinal surgery:
   - Instrumented surgery <2 Year
   - Discectomy <1 year
3. Spinal stenosis: Proven on MRI with significant impact on quality of life
4. Cervical myelopathy MRI proven
5. Foot drop with radicular pain < 6 months duration

Rheumatology

1. Known rheumatological disease (GSTT) or suspected (KCH) e.g. RA/Spondyloarthritis
2. Spinal pain & 4 or 5 of the below (ASAS)
   - Insidious onset
   - Onset <40 yrs old
   - Pain improves with activity
   - Worse with rest
   - Night pain improved with getting out of bed
3. Known osteoporotic fracture
4. Known or suspected connective tissue disease e.g. SLE or Ehlers Danlos with vascular symptoms
5. Metabolic bone disease - Paget's/OP

Hip

Physiotherapy

1. Tendinopathy - no previous physio OR previous good response
2. Trochanteric bursitis
3. Soft tissue injury
4. Mechanical hip/groin pain - no previous physio OR previous good response
5. Rehab post fracture

MCATTS

1. Tendinopathy & failed physio
1. Trochanteric bursitis & failed physio

(INTEGRATED CLINIC if GSTT)

1. Soft tissue injury with suspicion of tear
2. Mechanical hip/groin pain & failed physio
3. History of paediatric pathology - Perthes/SUFE/CDH

Orthopaedics

1. Previous surgery
   - Arthroplasty
   - Other surgery within 1 year
2. Severe OA on x-ray & failed physio
3. Known AVN head of femur

Rheumatology

1. Known rheumatological disease (GSTT) or suspected (KCH) e.g. RA/Spondyloarthritis
2. Known or suspected connective tissue disease e.g. SLE or Ehlers Danlos with vascular symptoms
3. Suspected Polymyalgia Rheumatica

General Surgery

1. Known hernia

ADVISE REFERRER

- Paediatrics (<16 yrs old)
- New fractures
- Lumps and bumps
- Spine pain post recent significant trauma (not WAD)
- Incomplete referrals
- Duplicate referrals
- Suspected RA/Inflammatory pain- vet directly to early inflammatory pathway GSTT
- Wrist ganglion/lump (excluding hand or finger lumps)

DISCUSS

- Any referral you are unsure about
- Any red flags not mentioned
- Investigation results you do not understand
- Anyone you feel requires Pain Management or Neurology referral

Knee

Physiotherapy

1. Anterior knee pain (PFJ integrated clinic if GSTT)
2. Known degenerate meniscal tear - no previous physio OR previous good response
3. Mechanical knee pain no previous physio OR previous good response
   e.g. tendinopathy, bursitis
4. Soft tissue injury
5. Early or moderate OA +/- previous injection
6. Severe OA documented not suitable for surgery

(Items 5–6: OA induction group if GSTT)

MCATTs

1. AKP not improved with physio (PFJ integrated clinic if GSTT)
2. Mechanical knee pain & failed physio (NOT AKP)
   e.g. tendinopathy, bursitis
3. Confirmed/suspected degenerate meniscal tear & failed physio

(Items 2–3: INTEGRATED CLINIC if GSTT)

4. Early to moderate OA & failed Physio / unable to tolerate exercise due to pain
   - possibly for injection

(Item 4: INTEGRATED CLINIC if GSTT)

5. Knee trauma with effusion (not imaged)
6. Soft tissue injury with suspicion of tear
   e.g. ACL, PCL, collaterals
7. Suspected meniscal tear
8. Recurrent locking knee
9. OA unknown severity or impact on QoL or function

Orthopaedics

Urgent

1. Acute locked knee
2. MCL or LCL injury (high grade)

Routine

1. Previous surgery
   - Arthroplasty
   - Other surgery within 1 year
2. New ACLD confirmed on MRI
3. Confirmed meniscal tear in patient <35
4. Severe OA & previously failed physio
5. Moderate OA with significant functional limitation & previously failed physio & injection
6. Recurrent knee/PFJ dislocations

Rheumatology

1. Known rheumatological disease (GSTT) or suspected (KCH) e.g. RA/Spondyloarthritis
2. Known or suspected connective tissue disease e.g. SLE or Ehlers Danlos with vascular symptoms
3. Confirmed knee synovitis

Shoulder & Elbow	Wrist/Hand	Foot/Ankle

Shoulder & Elbow

Physiotherapy:

1. Mechanical shoulder pain without night pain OR pain at rest
2. Mechanical elbow pain *e.g. bursitis*
3. 1st time shoulder dislocation – no trauma & no neurology OR post trauma and have been seen in A&E/MIU
4. Rehab post fracture
5. Rehab of multidirectional dislocation

MCATTS

INTEGRATED CLINIC if GSTT (items 1–3 below):

1. Shoulder capsulitis – no previous physio OR injection
2. Mechanical shoulder pain & failed physio
3. Confirmed partial thickness cuff tear
3. Proven full thickness rotator cuff tear over 75 yrs old
4. Shoulder pain with:
   - Pain at rest
   - Night pain
5. Shoulder pain + failed injection (NOT capsulitis)
6. Suspected cuff tear
7. Mechanical elbow pain - failed physio or injection

Orthopaedics

Urgent

1. Shoulder dislocation with neurology
2. Proven full thickness rotator cuff tear under 60 yrs old

Routine

1. Previous surgery
   - Arthroplasty
   - Other surgery within 1 year
2. 1st time shoulder dislocation & no neurology
3. Recurrent shoulder dislocations
4. Capsulitis - failed physio or injection
5. Known OA GHJ or ACJ failed conservative Rx and injection
6. Proven full thickness rotator cuff tear 60 - 75 yrs old
7. Known AVN humeral head

Rheumatology

1. Known rheumatological disease (GSTT) or suspected (KCH) e.g. RA/Spondyloarthritis
2. Known or suspected connective tissue disease e.g. SLE or Ehlers Danlos with vascular symptoms
3. Suspected Polymyalgia Rheumatica
4. Confirmed shoulder synovitis

Widespread pain

Non complex medical history to physiotherapy

Complex medical history to MCATTS

Please do not vet suspected Fibromyalgia to Rheumatology- complete WPI And SS score in clinic

Wrist/Hand

Hand Therapy GSTT /Physio VHG Kings

1. OA MCP / IP / 1st CMC / ST joint / RC joint
2. De Quervain's tenosynovitis - no previous treatment
3. Soft tissue injury with normal X-ray
4. Wrist tendinopathy
5. Wrist pain – atraumatic
6. Carpal Tunnel Syndrome
   - Sx only at night OR
   - intermittent symptoms during the day

   (CTS clinic if GSTT)
7. Ulnar neuropathy (elbow/wrist)
   - Sx only at night OR
   - intermittent symptoms during the Day

MCATTS

1. OA MCP / IP / ST / RC joint & failed hand therapy
2. Soft tissue injury & failed hand therapy
3. Wrist trauma > 6/52 post injury
4. Wrist pain atraumatic & failed hand therapy
5. Ulnar neuropathy (elbow/hand)
   - not responding to splinting
6. Carpal tunnel Syndrome
   - No wasting, no weakness
   - Mildly +ve NCS
   - Day symptoms

   (CTS clinic if GSTT)

MCATTs Injector

1. Trigger finger/thumb
2. De Quervain's & failed Hand Therapy OR requesting injection
3. Mild/moderate OA 1st CMC
4. Carpal tunnel not responded to activity modification and splinting (CTS clinic if GSTT)

Hands and Plastics [Orthopaedics at KCH]

Urgent

1. Hand trauma < 6/52
2. Wrist trauma < 6/52

Routine

1. Trigger finger/thumb & failed x2 injections
2. Carpal tunnel syndrome & any of the below:
   - Moderate/severe on NCS
   - Constant sensory loss
   - Motor loss thumb abduction
   - Mild to mod Sx & failed conservative management >4/12
3. De Quervain's tenosynovitis & failed x2 injections
4. OA 1st CMC / ST / RC / MCP / IP & failed conservative Mx including injection
5. Ulnar neuropathy (elbow/wrist)
   - Motor loss / clawing
   - Constant sensory loss
   - Significant wasting
7. Dupuytren's Contracture
8. Seed ganglion or mucous cyst > 30˚

Rheumatology

1. Known rheumatological disease (GSTT) or suspected (KCH) e.g. RA/Spondyloarthritis
2. Known or suspected connective tissue disease e.g. SLE
3. Confirmed wrist / hand joint synovitis
4. Dactylitis

Sports Clinic

Sporting injuries only

Must be Impacting baseline level of sport participation

Patient must do sport/intense exercise >3 days per week?

Foot/Ankle

Physiotherapy

1. Tendinopathy / tendonitis / tendinosis / fasciopathy – no previous Physio OR previous good response

   e.g. plantarfasciitis, tendinopathy, tib post dysfunction , bursitis
2. Ankle instability – no previous Physio OR previous good response
3. Acute soft tissue injuries - *e.g. calf tear*
4. Medial tibial stress syndrome
5. Ankle impingement - no previous Physio OR previous good response
6. Early/moderate OA ankle/foot

MCATTS

INTEGRATED CLINIC if GSTT (items 1–3 below):

1. Tendinopathy / tendonitis / tendonosis / chronic ankle instability & failed Physio
2. Sport injuries & failed Physio
3. Unspecified mechanical foot/shin/ankle pain
3. Post traumatic foot pain > 6/12 / ankle impingement / plantar fasciopathy & failed Physio
4. Degenerative tendon tear - *e.g. tib post*
5. Tib post dysfunction with deformity
6. Morton's neuroma
7. Metatarsalgia/bursitis
8. Peripheral neural entrapment /neuropathy

Orthopaedics

Urgent

1. New tendon rupture
2. Confirmed tendon tear post trauma – *e.g. peroneal split, tib post*

Routine

1. Foot and ankle pain related to previous surgery
2. Hallux valgus
3. Severe OA & failed physio
4. Known Gr 4 tib post dysfunction
5. Suspected stress fracture proven on imaging
6. Plantar plate disruption proven on imaging
7. Specific surgical appliance needs

Rheumatology

1. Known rheumatological disease (GSTT) or suspected (KCH) e.g. RA/Spondyloarthritis
2. Known or suspected connective tissue disease e.g. SLE or Ehlers Danlos with vascular symptoms
3. Dactylitis
4. Confirmed ankle/foot synovitis

Orthotics

For referrals asking for orthotics please advise referrer to direct patient to self refer to foot health or refer directly to podiatry

GSTT Physiotherapy Vetting

Urgency

Urgent	Routine
• Acute injury no marked improvement in 3 weeks<br>• 1st episode of LBP not improved in 6 weeks<br>• Post surgery<br>• Post plaster cast removal<br>• Post fracture<br>• Off work due to *this* problem<br>• Sleep affected due to *this* problem<br>• Recent onset worsening radiating pain or sensory symptoms<br>****Worsening weakness to MCATS****<br>• Significantly worsening symptoms with no apparent cause	All other referrals

GSTT Sports Clinic

Inclusion	Exclusion
• Borough of Lambeth & Southwark patients<br>• Acute or persistent Musculoskeletal injury or condition preventing sporting activity or impairing participation<br>• Must have a restriction from their specific sports or high level functional activity<br>• Must be of moderate-high activity (150 minutes p/w) levels (WHO guidance)<br>• Failed previous rehabilitation<br>• Would benefit from a specialist sports opinion<br>• Current physiotherapy patients with spinal pain from sports not progressing & needing specialist opinion	• Children under 18 years of age<br>• Non- musculoskeletal pain<br>• Conditions where already established MSK pathway: Patellofemoral joint pain syndrome, Spinal & radicular pain presentations<br>• Fractures<br>• General MSK conditions in low activity population group or OA in more active population<br>• Widespread multi-site chronic pain patients<br>• Patients suitable for Acute Orthopaedic Clinics- “hot knee/shoulder” etc.<br>• ACL/Meniscal/Dislocations injuries needing Orthopaedic input<br>• Sedentary/ low activity level patients as per WHO guidance
